# Seasonal Influenza Vaccination in People who Have Contact With Birds

**DOI:** 10.1111/irv.70101

**Published:** 2025-04-19

**Authors:** Amy Thomas, Suzanne Gokool, Harry Whitlow, Genevieve Clapp, Peter Moore, Richard Puleston, Louise E. Smith, Riinu Pae, Ellen Brooks‐Pollock

**Affiliations:** ^1^ Population Health Sciences, Bristol Medical School University of Bristol Bristol UK; ^2^ Bristol Veterinary School University of Bristol Bristol UK; ^3^ UKHSA, UK Health Security Agency London UK; ^4^ NIHR Health Protection Research Unit in Behavioural Science and Evaluation University of Bristol Bristol UK

**Keywords:** seasonal influenza, vaccination, influenza, public health policy, vaccination, zoonotic influenza

## Abstract

**Background:**

Following the 2021–2022 avian influenza panzootic in birds and wildlife, seasonal influenza vaccines have been advised to occupationally high‐risk groups to reduce the likelihood of coincidental infection in humans with both seasonal and avian influenza A viruses.

**Methods:**

We developed and launched a questionnaire aimed at poultry workers and people in direct contact with birds to understand awareness and uptake of seasonal influenza vaccination. We collected responses in‐person at an agricultural trade event and online.

**Findings:**

The questionnaire was completed by 225 individuals from across the United Kingdom. The most commonly reported reason for vaccination was protection against seasonal influenza (82%, 63 of 77). Nearly, all individuals aged ≥65 years reported that the vaccine was recommended for them (24 of 28). There was no difference in recommendation for occupational groups. Most vaccinees were aged over 60 years (60%, 29 of 48); however, coverage was lower than expected in the ≥ 65 target group. Vaccination in those exposed to avian influenza was low (32%, 9 of 28). Not having enough time was the single most reported reason for not getting vaccinated in those intending to. Individuals unintending to be vaccinated perceived natural immunity to be better than receiving the vaccine as well as lack of awareness and time.

**Conclusions:**

Our findings suggest that targeted campaigns in occupationally exposed groups need to be undertaken to improve communication of information and access to vaccine clinics. We recommend co‐production methods to optimise this public health strategy for increased knowledge and future vaccine uptake.

## 1 Introduction

Influenza is a globally important pathogen with a high pandemic potential. Of the four influenza virus types, influenza A (subtypes H1N1, H2N2 and H3N2) and B circulate in the human population and cause seasonal influenza epidemics, usually in the winter in temperate regions [1]. The high pandemic potential of influenza is due to the ability of distinct subtypes to reassort either in human or porcine hosts and create novel influenza subtypes, against which there is little or non‐existing prior immunity in the human population (antigenic shift) [2, 3]. Recent increases in avian influenza (AI) outbreaks in Europe and America have heightened concern about the risk of a novel virus emerging.

Wild birds, primarily waterfowl, are considered a natural reservoir for influenza A viruses, where all subtypes circulate in the low pathogenic form, causing limited or no symptoms [4]. However, influenza A subtypes (H5 and H7) are considered highly pathogenic AI (HPAI) because of rapid transmission, serious infection and death in domestic and wild birds [5, 6]. In recent years, there has been an increase in HPAI A(H5N1) influenza outbreaks in wild and domestic birds. In 2021–2022, 48 million birds were culled due to HPAI across Europe, with many further deaths in wild birds from infection [7]. Transmission of HPAI A(H5N1) clade 2.3.4.4b in wild birds continued throughout summer 2022 in Great Britain and northwest Europe, unlike previous seasons [8]. Furthermore, there have been increased reports of infection in wild mammals during this panzootic, with evidence of genomic changes to support an advantage for mammalian infection and transmission [9]. In the United States, recent widespread transmission in cattle and isolated cases of cattle‐to‐human transmission has raised concerns that further transmission could occur to humans, leading to epidemics or a pandemic and resulting in heightened public health risk assessments [10, 11, 12].

Most cases of HPAI in humans occur following very close contact with infected birds and range in severity. Recent cases have mostly been asymptomatic or mild and self‐limiting. However, infection with other strains has been serious or fatal [13]. In the United Kingdom, public health action is initiated for suspected or confirmed AI infections in birds to prevent human infection, including advice and training on appropriate use of PPE, information about HPAI and possible follow‐up and prophylactic antiviral treatment for people who have been exposed [14]. Following pilot asymptomatic swabbing of people following exposure to confirmed HPAI A(H5N1) in England, [15] an asymptomatic surveillance programme was commenced by UK Health Security Agency (UKHSA). In England, AI detections in birds are confirmed by Animal and Plant Health Agency (APHA), and UKHSA is notified to investigate exposed persons. Between March 2023 to 10 July 2023, 144 exposed persons from eight infected premises were tested, of which four were positive for H5N1 (clade 2.3.4.4b). However, the significance of these detections cannot always be certain—at least two were likely contamination and not true infection [16].

In the United Kingdom, seasonal influenza vaccination has been recommended since the late 1960s to protect clinical groups at higher risk of severe illness. In 2000, the programme was extended to all individuals over 65 years [17] and has been further extended to pregnant women, those caring for people with a weakened immune system, and front line health and social care workers; this is similar to many European countries [18]. In 2013, the programme was extended to healthy children aged 2 to less than 18 years old using the live attenuated influenza vaccine (LAIV). All vaccines in the UK programme are quadrivalent—two influenza A subtypes and both lineages of influenza B (Victoria and Yamagata) [17]. Some European countries recommend seasonal influenza vaccination for individuals occupationally exposed to animals infected with AI [19]. In 2023 in the United Kingdom, the Joint Committee on Vaccination and Immunisation (JCVI) advised that workers in the poultry and avian animal health industries should be considered for the seasonal inactivated influenza vaccine [17].

For individuals at high risk of AI exposure, the motivation for vaccinating with the seasonal influenza vaccine is because the vaccine reduces the risk of infection and decreases viral load of seasonal influenza strains. This therefore minimises the risk of human influenza and AI coinfection and subsequently reduces the risk of reassortment events [19]. In addition, while seasonal influenza vaccines do not provide specific protection against AI, there may be some level of cross‐protective immunity via cross‐reactive neutralising antibody and T‐cell responses [20]_._ In older (≥ 60 years) European adults, a low but significant heterosubtypic seroconversion against H5N1 and H9N2 following seasonal trivalent inactivated influenza vaccine (with 2006–2007 H1N1 and H1N1pdm09 subtypes) has been shown [21].

Here, we report on awareness and attitudes to seasonal influenza vaccination among people who have contact with birds in the United Kingdom. We used data from the Avian Contact Study, a codeveloped questionnaire aimed at poultry workers and individuals in contact with any type of bird(s). Our findings aim to inform discourse on including this high‐risk group in future seasonal influenza vaccination programmes.

## 2 Methods

### 2.1 Brief Description of the Avian Contact Study

The Avian Contact Study aimed to inform and optimise current zoonotic influenza public health measures. A questionnaire was co‐produced with members of the poultry industry, and in consultation with UKHSA, Animal Plant Health Agency and the Health Protection Research Unit (HPRU) in Behavioural Science and Evaluation to understand awareness and potential transmission of HPAI among people who have contact with domestic or wild birds such as poultry farmers and bird‐keepers. A questionnaire was developed and deployed to collect information on the type of contact people have with birds, their social contacts, perceptions of AI generally and perceptions of their own personal health. Full details of the questionnaire methodology can be found in the Data Note [22] and the questionnaire is available as *Extended data* [23]. We present here the results relating to one element of the Avian Contact Study questionnaire on vaccination.

### 2.2 Ethics and Consent

Ethical approval for the study was obtained from the University of Bristol, Faculty Research Ethics Committee, approval number 17048 on 16 January 2024.

Informed written consent (using e‐consent hosted on Research Electronic Data CAPture tools, REDCap) for the use of data collected via the questionnaire was obtained from respondents.

### 2.3 Data Collection

The questionnaire was initially delivered in‐person at the British Pig and Poultry Fair (15–16 May 2024) and was subsequently circulated online via various poultry forums and networks via word‐of‐mouth. All questions were optional. Data captured between 15 May 2024 and 31 July 2024 were included in these analyses. Questionnaire data were collected using REDCap [24], hosted at the University of Bristol. Data were exported from REDCap to R studio (version 4.4.0) for data manipulation and analysis.

### 2.4 Demographic and Background Questions

We collected background demographic data on age, gender, occupation and current levels of health.

### 2.5 Influenza Vaccination

The following vaccine‐related questions were included in the Avian Contact Study questionnaire:

1. Has the seasonal (winter) flu vaccine provided by the NHS been recommended for you?
  ○ Yes/No/I do not know.
2. Have you been vaccinated against seasonal (winter) flu in the last 12 months?
  ○ Yes as provided by the NHS.
  ○ Yes, I paid for it myself.
  ○ No, but I intend to get vaccinated.
  ○ No, I am undecided.
  ○ No, I am not intending to get vaccinated.
  ○ I do not know.
3. [If vaccinated] When were you vaccinated?
4. [If vaccinated] What factors influenced your decision to get vaccinated?
  ○ For protection against seasonal (winter) flu.
  ○ Convenience of appointment times.
  ○ Convenience of vaccine centre location.
  ○ The vaccine is safe—no side effects.
  ○ The vaccine is effective at reducing the risk of seasonal (winter) flu.
  ○ Other people I know are vaccinated.
  ○ The vaccine has been recommended for me.
  ○ For protection against avian influenza (bird flu).
  ○ To protect other people.
5. [If not vaccinated/not planning to get vaccinated/undecided] What factors have prevented you from getting vaccinated?
  ○ I have not had time to get vaccinated.
  ○ I do not know where to get vaccinated.
  ○ It is too far/inconvenient to get vaccinated.
  ○ The vaccine is too expensive.
6. [If not vaccinated/not planning to get vaccinated/undecided] What factors influenced your indecision or decision to not get vaccinated?
  ○ The vaccine is too expensive.
  ○ I am not sure if the vaccine is effective.
  ○ The vaccine may cause short‐term side effects.
  ○ The vaccine may cause long‐term side effects.
  ○ I am not likely to catch seasonal (winter) flu.
  ○ I will not get very ill if I catch seasonal (winter) flu.
  ○ I am allergic to the vaccine.
  ○ It is better to get natural immunity.
  ○ Other.
    - [If Other selected] Please state what factors influenced your indecision or decision to not get vaccinated (free text).

### 2.6 Data Analysis

Respondents who reported information for at least one of the five vaccine questions were included in the analyses, that is, if respondents did not answer at least one of the five vaccine‐related questions, their record was excluded from analyses. The proportion of individuals responding to each question was calculated and reported alongside 95% confidence intervals for a single proportion using the two proportions z‐test, as well as raw counts. Responses were grouped by age, gender and occupation. Occupations with fewer than five individuals per category are not shown to preserve anonymity. We calculated seasonal influenza vaccine uptake for individuals based on their eligibility to receive the vaccine via the NHS guidance of individuals ≥ 65 years old. We further considered an ‘at‐risk’ group defined as individuals reporting exposure to AI virus. We could not reliably identify occupationally at‐risk individuals as per the Green Book guidance (poultry and related workers) due to ambiguity in occupational risk [17]. Individuals < 65 years old and who did not report AI virus exposure were grouped as ‘no risk’. We grouped responses for Question 2 (vaccination status) into four categories: Yes (Yes as provided by the NHS or Yes I paid for it myself); No (No I am not intending to get vaccinated); Intending (No but I intend to get vaccinated); and Do not know (I do not know or No I am undecided). For responses of ‘Other’ given in Question 6 (factors influencing indecision/decision to not get vaccinated), we grouped free text responses into six main themes/reasons: Does not want it; Not aware; No time; Not offered; Could not access from GP and Forgot.

## 3 Results

**FIGURE 1:**
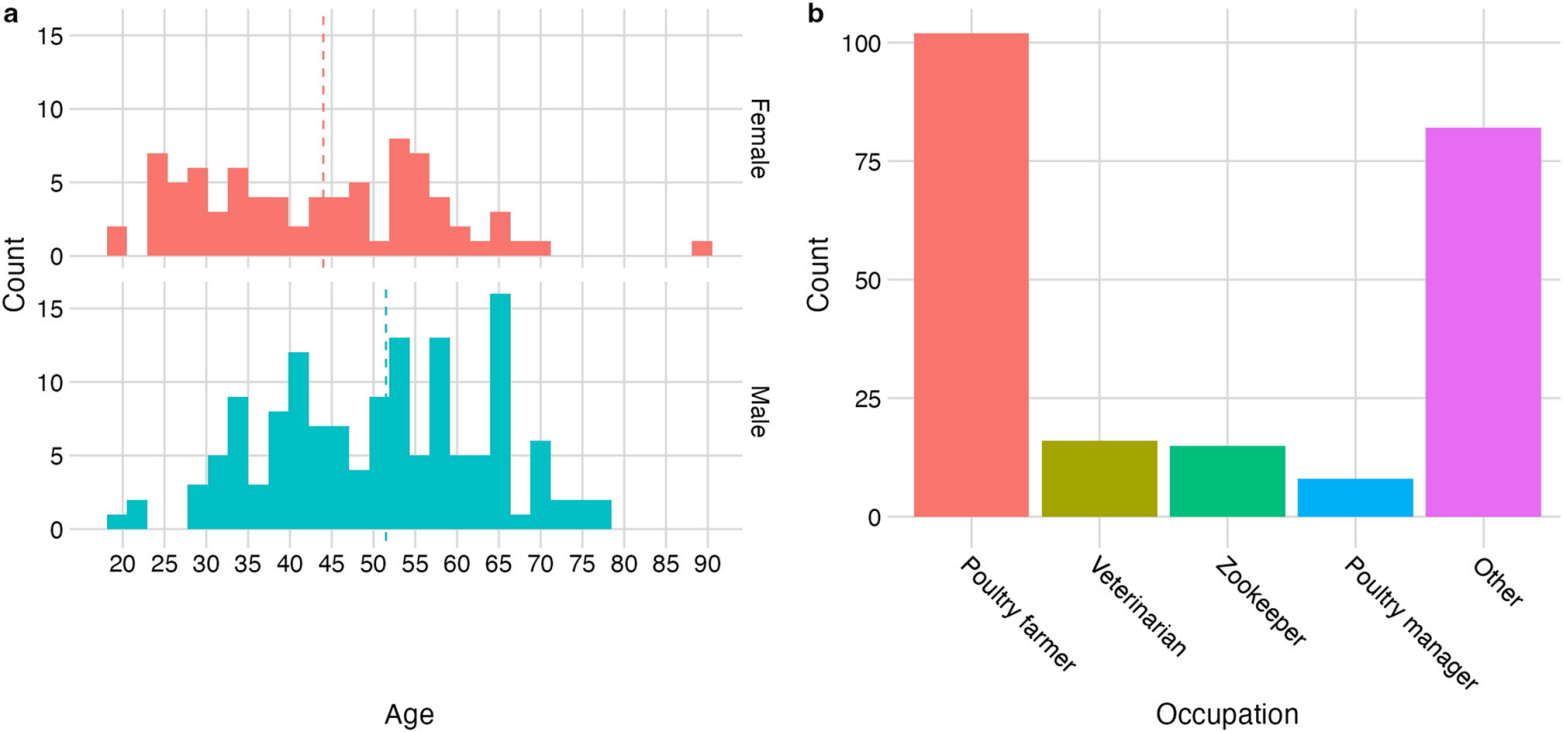
Distribution of respondent age, gender and occupation (a) Age distribution of respondents grouped by gender—female in red and male in blue (note one ‘Other’ and one NA response not plotted). (b) Top 4 occupations reported (occupations with fewer than five observations grouped with ‘Other’).

A complete description of methods, public involvement and participant demographics can be found in the Data Note [22]. A total of 225 people took part in the study between May to July 2024, two respondents were excluded from all analyses as they did not provide responses to at least one vaccine question, and one participant did not complete the gender question. Briefly, most respondents were male (63%, 140 of 222; female 37%, 82 of 222) and between 30 to 59 years (69%, 153 of 222). Male respondents had a higher median age than female respondents (52 compared to 44 years) (Figure 1a). Poultry farmer was the most frequently reported occupation (72%, 102 of 222), followed by veterinarian, zookeeper and poultry manager (Figure 1b).

## 4 Seasonal Influenza Vaccination

### 4.1 Eligibility for Free Seasonal Influenza Vaccination

**FIGURE 2:**
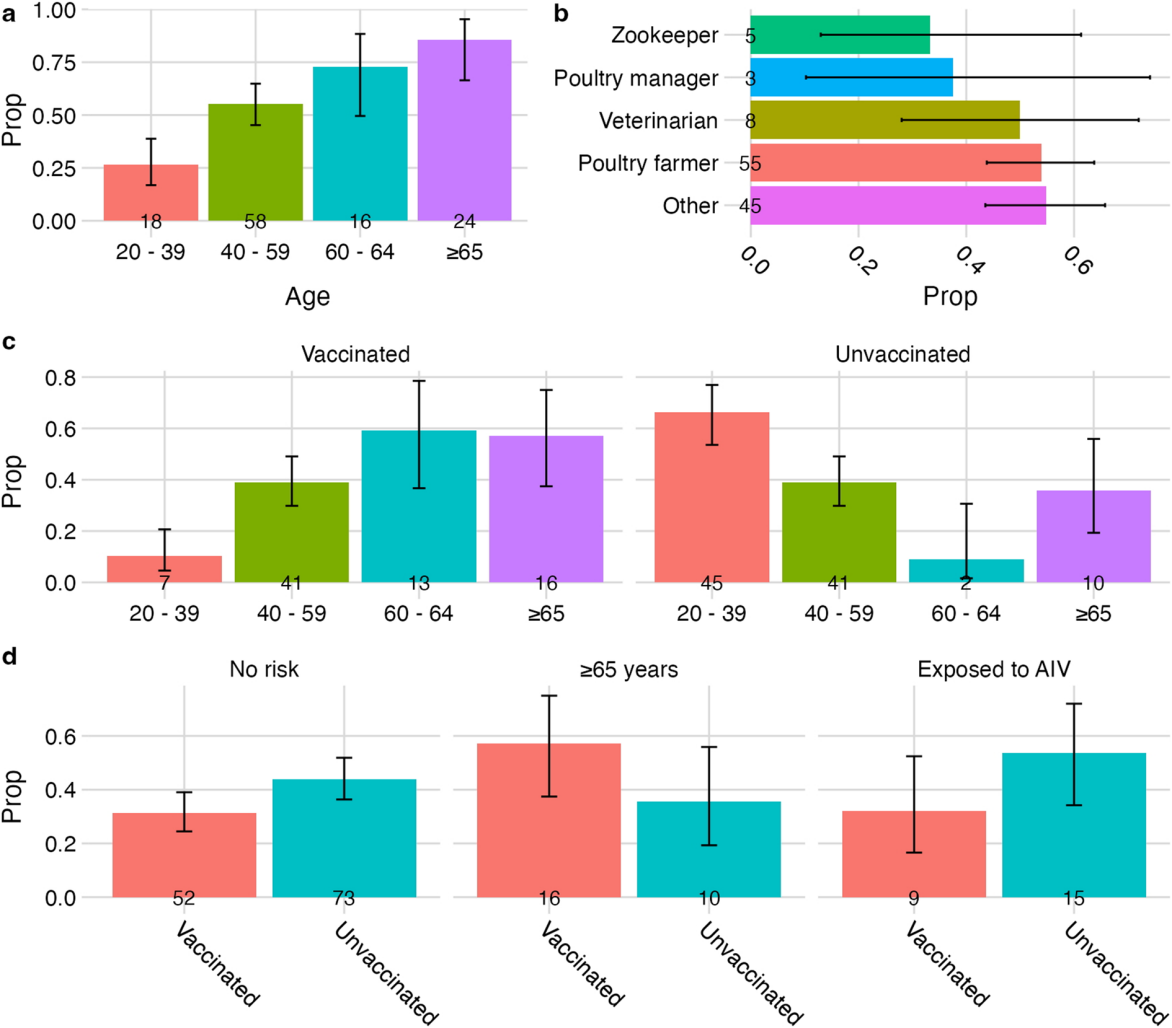
Seasonal influenza vaccine recommendation and uptake. (a) Proportion self‐reporting recommendation to receive seasonal influenza vaccine by age. (b) Proportion self‐reporting recommendation to receive seasonal influenza vaccine by occupation. (c) Proportion of individuals receiving seasonal influenza vaccine in the last 12 months by age. (d) Proportion of individuals receiving seasonal influenza vaccine in the last 12 months by eligibility/risk group. Those considered ‘at‐risk’ reported being exposed to avian influenza virus (AIV). Error bars represent 95% confidence intervals for single proportion.

Across all age groups, 52% (116 of 222) of individuals reported that the seasonal influenza vaccine was recommended for them, 4% did not know. The proportion of individuals reporting recommendation increased with age (Figure 2a). Nearly, all individuals aged 65 years and over‐reported that the vaccine had been recommended for them (86%, 24 of 28) but less than half of under 65s reported recommendation (47%, 92 of 195). There was no evidence of a difference in recommendation among commonly worked occupations (Figure 2b). Taken together, this suggests low awareness of the vaccine eligibility criteria for workers in the poultry and avian animal health industries to receive seasonal influenza vaccine.

### 4.2 Trends in Seasonal Influenza Vaccine Uptake

Overall, 35% of respondents (77 of 223) had been vaccinated against seasonal influenza, of these 79% (61 of 77) had the vaccine provided by the NHS and a further 21% paid for the vaccine themselves (16 of 77). Most individuals did not intend to receive the vaccine (44%, 98 of 223), while 11% (25 of 223) had not been vaccinated but intended to and 10% (22 of 223) did not know. Seasonal influenza vaccine uptake increased with increasing age; the greatest proportion of vaccinees (60%, 29 of 48) were aged ≥ 60 years (Figure 2c). Conversely, younger age groups were less likely to be vaccinated, with the largest unvaccinated proportion (66%, 45 of 68) aged between 20–39 years (Figure 2c).

We considered vaccine uptake based on eligibility and risk. Given our sample, 28 of 223 respondents were ≥ 65 years old and eligible to receive seasonal influenza vaccine as per the UK seasonal influenza vaccine campaign [17]. Of these, just over half had been vaccinated in the previous 12 months (57%, 16 of 28). Vaccine uptake in those reporting AI virus exposure (32%, 9 of 28) was lower than the ≥ 65‐year group but marginally higher than the no‐risk group (31%, 52 of 166) (Figure 2d).

### 4.3 Reasons for Seasonal Influenza Vaccination

**FIGURE 3:**
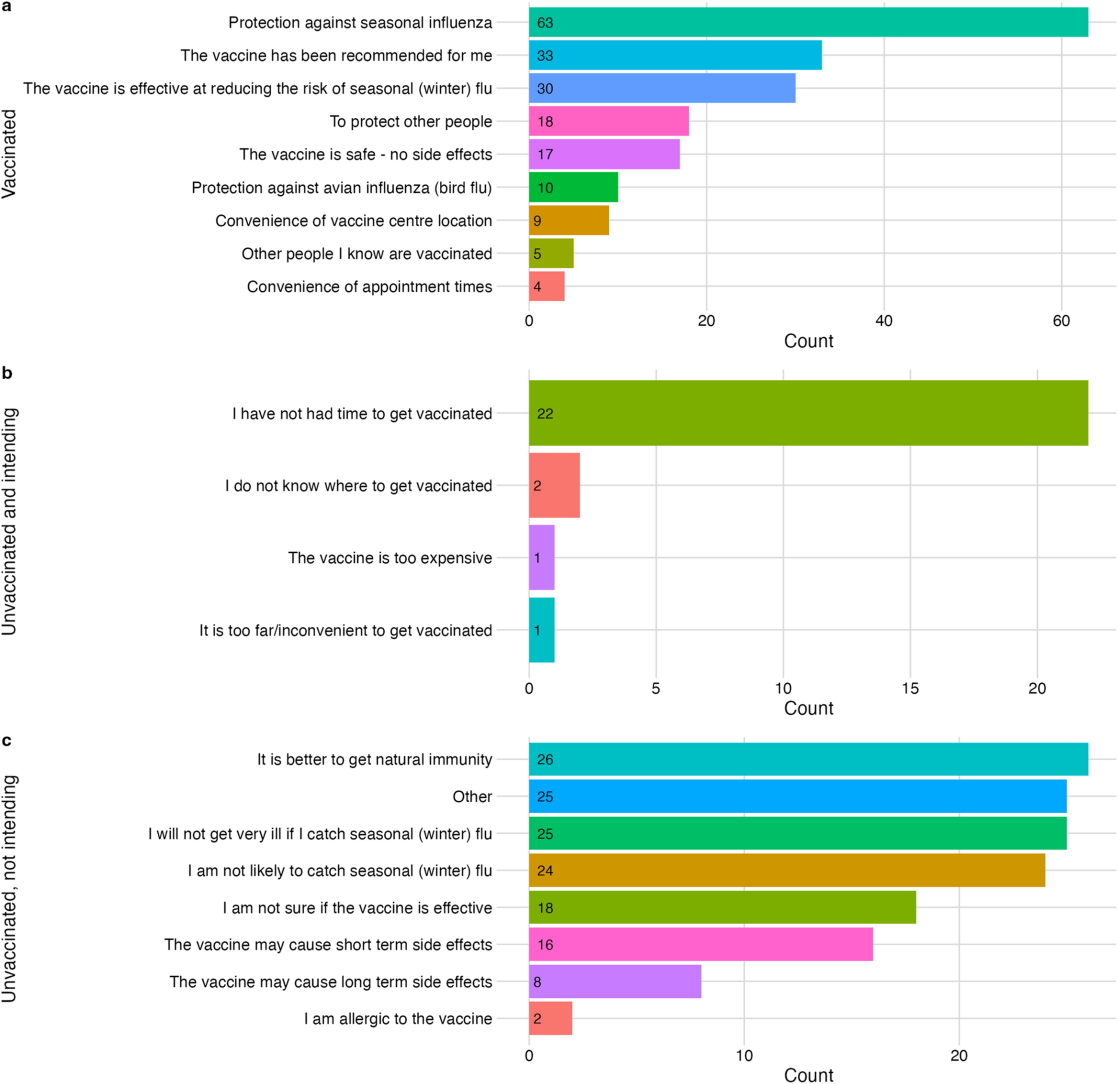
Reasons for seasonal influenza vaccination. (a) reasons for receiving the seasonal influenza vaccine among vaccinated individuals, (b) reasons preventing an individual from becoming vaccinated among individuals intending to be vaccinated, (c) factors influencing indecision or decision to not be vaccinated among those not intending to be vaccinated.

Among those who were vaccinated, the most commonly reported reason for vaccination was protection against seasonal influenza (82%, 63 of 77). Protection against AI, convenience of getting vaccinated (location/appointment time) and the behaviour of others were infrequently reported as reasons for being vaccinated (Figure 3a).

Among individuals who intended to get vaccinated, not having enough time to get vaccinated was the single most reported reason for not having the seasonal influenza vaccine (88%, 22 of 25; Figure 3b).

Among individuals who were not intending to get vaccinated, frequently reported reasons for not getting vaccinated were perceiving that it was better to get natural immunity than to receive the vaccine (27%, 26 of 98), and thinking that seasonal influenza would not make them very ill if they did catch it. Short‐ or long‐term side effects were less frequently reported reasons. Of the respondents answering ‘Other’ (25 of 98), 21 proceeded to provide free text detail on why they were not intending to get vaccinated. These responses were coded into six main themes/reasons that reflected largely not wanting the vaccine (‘Doesn't want it’; 6 of 21), or that they were ‘Not aware’ (6 of 21) or had ‘No time’ (4 of 21). ‘Not offered’ (3 of 21), ‘Couldn't access from GP (1 of 21) and “Forgot”’ (1 of 21) were also given (Figure 3c).

## 5 Discussion

Seasonal influenza vaccination is an important public health measure aimed at limiting influenza‐related morbidity and mortality. Although not intended for protection against AI infection, recent increases in AI in birds and mammals have increased the risk of human–AI co‐infection and reassortment. As a result, poultry keepers and others at high risk of AI infection have become eligible in seasonal influenza vaccine programmes. The analysis presented here suggests that awareness and uptake is low in individuals at high risk of AI exposure.

Self‐reported reasons for not being vaccinated, including not having time or knowing where to get vaccinated, highlighted the need for accessible and convenient vaccine centres, especially in rural areas. The Fit2Farm survey found that UK farmers work long hours (an average of 65 h a week compared to the UK national average of 37) and have limited days off [25]; therefore, accessing vaccine centres may be challenging. In particular, the Fit2Farm survey revealed that almost half of 700 UK respondents said they were able to take a regular day off only once or twice a month, with a quarter reporting 1 to 2 days off a year. Further, male farmers, which make up approximately 80% of our agricultural workforce (holders and managers) in England [26], are perceived to be a ‘hard to reach’ group in relation to engaging with health promotion interventions and preventive health behaviours [27]. Efforts to improve access to healthcare for farmers include outreach programmes and nurse‐led services. One example is a pop‐up site where the influenza vaccine was available; one farmer reported this initiative's success in encouraging vaccination was due to saving time from visiting a GP surgery and avoiding the need to change clothes to do so [28]. Further, Nye et al. identified that a constant presence of services such as pop‐up hubs build‐up trust, and this is important for encouraging new help‐seeking behaviours, but even infrequent pop‐ups were productive in gaining participation [28]. Our results support the need for flexible vaccine delivery models that are tailored to meet the demands of individuals working in poultry and farming related occupations [29]. The importance of these agile systems has also been highlighted for delivery of antivirals to those exposed to AI [30].

The WHO and EU target for influenza vaccine coverage rate is 75% in target groups. In our sample, those receiving the vaccine from the NHS were mostly older individuals aged ≥ 60 years to prevent them from seasonal influenza (60%). Since 2020, the estimated uptake in individuals aged 65 and over in England is between 78% and 82%. Prior to the COVID pandemic, uptake ranged between 70% and 74% [31]. Here, we report lower coverage, with just over half of target individuals aged ≥ 65 years (57%) having been vaccinated, albeit the sample size is small, reflecting the need to create vaccine strategies that are suitable to this demographic.

We identified a miscommunication or lack of understanding regarding why individuals in contact with birds are recommended to receive seasonal influenza vaccine. Non‐vaccinated individuals reported that they thought it was better to obtain natural immunity, and protection against AI was not a commonly cited reason for vaccination. Furthermore, participants who come into occupational contact with poultry were not aware of the advice for seasonal influenza vaccination from the 2023 JCVI. This highlights the need to enhance engagement activities with this group for improved communication in developing and delivering vaccination campaigns.

Co‐production and co‐creating methodologies can be effective in engaging with members of the farming community to undertake zoonotic disease research [32] and can offer a method for co‐producing interventions with the community [33]. It also builds trust and working relationships between farming communities and organisations, making future co‐production easier. For example, this was an outcome of a recent project in which public health advice was co‐produced with those who have worked on premises infected with HPAI [34]. The Fit2Farm survey identified that farmers participating in nonfarming activities are likely to be participating in farm network discussion groups [25] so targeting these groups for patient and public involvement and engagement (PPIE) activities, alongside visiting agricultural events such as livestock auctions [28], are viable routes for engaging with members of the farming community.

The rationale for JCVI's vaccine consideration for this high‐risk group is to prevent reassortment of the virus [17]. However, several studies have reported non‐specific protection against AI in individuals vaccinated with the seasonal influenza vaccine, albeit with potentially limited usefulness in terms of longevity and functional activity of responses [20, 35, 36]. Further work is needed to characterise the benefit for individuals repeatedly exposed to AI, such as through occupational exposure. Approximately 18.5 million influenza vaccines are given each year in England; including occupationally exposed individuals represents a small addition, representing less than 0.5% [37].

The Avian Contact Study questionnaire provides a timely and current snapshot of attitudes and uptake relevant for planning seasonal autumn influenza vaccine campaigns for people in contact with poultry. Moreover, our approach provides information for members of the farming community who have historically been identified as vulnerable in terms of health and occupational needs [38] as well as experiencing barriers with primary healthcare providers [39]. We achieved good coverage of respondents in older age categories through in‐person and online questionnaire completion [22]. While validated measures investigating vaccine perceptions and hesitancy exist [40], these were not used due to time and space considerations for the questionnaire. We chose to limit free text‐responses where possible. This affected participants' ability to input other answers for multiple choice questions (e.g. ‘what factors influenced your decision to get vaccinated?’). Future iterations of the questionnaire should aim to include ‘Other’, and free text responses to allow participants to represent their views accurately. Our sample was composed of mostly poultry farmers and hobby bird‐keepers, and those in contact with wild birds are underrepresented; therefore, generalisation to other groups in contact with birds should be done cautiously. In‐person recruitment at an agricultural show may have excluded ‘front‐line’ poultry workers due to preferential attendance from managers and specialists in this field. Further, the questionnaire and promotion were conducted in English, possibly marginalising groups—many poultry subcontractors do not have English as their first language [41]. Reporting biases may also exist between online and in‐person responses. Interpretation of small sample sizes should be done with caution. Future studies should seek to address these data gaps by maximising recruitment across multiple groups and sectors.

## 6 Conclusion

We have highlighted the disconnect between policy and influenza vaccine implementation. There is a need to work with members of the poultry and wild‐bird community for informing public health responses. Current recommendations to vaccinate this cohort will be thwarted if tailored campaigns are not undertaken. We recommend using co‐production to identify methods for improving access to vaccine clinics and explore ways for effective communication.

## Author Contributions

**Amy Thomas:** conceptualization, data curation, formal analysis, funding acquisition, investigation, methodology, project administration, visualization, writing – review and editing, writing – original draft, software. **Suzanne Gokool:** investigation, methodology, project administration, writing – review and editing. **Harry Whitlow:** investigation, writing – review and editing. **Genevieve Clapp:** investigation, project administration, writing – review and editing. **Peter Moore:** investigation, methodology, writing – review and editing. **Richard Puleston:** investigation, methodology, writing – review and editing. **Louise E. Smith:** investigation, methodology, writing – review and editing. **Riinu Pae:** investigation, methodology, writing – review and editing. **Ellen Brooks‐Pollock:** data curation, project administration, supervision, visualization, writing – original draft, writing – review and editing, validation, resources.

## Conflicts of Interest

LES, RP and RPu are employees of the UK Health Security Agency. LES receives consultancy fees from the Sanofi group of companies and other life sciences companies. PM is an employee of the Animal Plant and Health Agency. The views expressed are those of the authors and not necessarily those of the UKHSA or the Department of Health and Social Care.

### Peer Review

The peer review history for this article is available at https://www.webofscience.com/api/gateway/wos/peer‐review/10.1111/irv.70101.

## Underlying Data

Repository data.bris: The Avian Contact Study: questionnaire data 15 May—31 July 2024. Data are openly available at the University of Bristol Research Data Repository (data.bris), at https://doi.org/10.5523/bris.3nmqsrbv5ruom2abn0ql6e8yh2 [42] .

This project contains the following underlying data:

- Data file 1. (Raw underlying questionnaire data—csv file)
- Data file 2. (Raw underlying questionnaire data—.RDS file)
- Data file 3. (Associated data dictionary—csv file)
- Data file 4. (Code for importing underlying data in csv format into R for setting up labelled data—.r file)
- Data file 5. (Blank consent form and participant information sheet—pdf file)

Data are available under the terms of National Archives' Non‐Commercial Government Licence for public sector information.

## Extended Data

Repository Zenodo: The Avian Contact Study Questionnaire and Data Dictionary [10.5281/zenodo.13617061]

This project contains the following extended data:

- AvianInfluenzaSocialContactSu.pdf (The final questionnaire REDCap—PDF)
- AvianInfluenzaSocialContactSur_DataDictionary_080824v1.csv (Associated data dictionary—csv file)

Data are available under the terms of the Creative Commons Attribution 4.0 International license (CC‐BY 4.0).

## Software Availability

Source code available from: https://github.com/amythomas/aviancontactstudy/tree/vaccination

Archived source code at the time of publication: https://doi.org/10.5281/zenodo.15002734 [43]

License: Creative Commons Attribution 4.0 International license (CC‐BY 4.0).
